# BIOLOGICAL AGING AND APOE STATUS REWIRE INTER-OMIC ASSOCIATIONS RELATED TO BIOENERGETICS IN HUMANS

**DOI:** 10.1093/geroni/igad104.2058

**Published:** 2023-12-21

**Authors:** Noa Rappaport, Dylan Ellis, Kengo Watanabe, Tomasz Wilmanski, Leroy Hood, Nathan Price, Cory Funk, Priyanka Baloni

**Affiliations:** Institute for Systems Biology, Seattle, Washington, United States; Institute for Systems Biology, Seattle, Washington, United States; Institute for Systems Biology, Seattle, Washington, United States; Institute for Systems Biology, Seattle, Washington, United States; Institute for Systems Biology, Seattle, Washington, United States; Thorne HealthTech, New York City, New York, United States; Institute for Systems Biology, Seattle, Washington, United States; Purdue University, West Lafayette, Indiana, United States

## Abstract

Apolipoprotein E (APOE) modifies human aging, with the ε2 and ε4 alleles being among the strongest genetic predictors of longevity and Alzheimer’s disease, respectively. However, the mechanisms of APOE’s impact on aging and cognition remain largely uncharacterized. In this study, we analyzed inter-omic context-dependent association patterns across APOE genotype, sex, and health in an undiagnosed cohort of 1950 individuals. We hypothesized that APOE genotypes would show variation in energy metabolites tied to previously-validated metrics of ’biological aging’, a modifiable health metric based on blood biomarkers. Our analysis identified top APOE-associated metabolites as diacylglycerols, including oleoyl- and linoleoyl-arachidonoyl-glycerols, similarly increased in APOE ε2- and ε4- carriers compared to ε3-homozygotes. Male ε2-carriers and biologically-older males displayed a similar increase in associations between insulin resistance and bioenergetic metabolites including pyruvate, glucose, gluconate, and lactate, a trend which was validated in an independent cohort of TwinsUK females. These results provide an atlas of APOE allele-rewired associations and support the involvement of bioenergetic pathways in mediating APOE impact on longevity and AD risk, suggesting targets for enhancing healthspan via lifestyle-modifications or drug-repurposing.

